# Biomanufacturing in low Earth orbit for regenerative medicine

**DOI:** 10.1016/j.stemcr.2021.12.001

**Published:** 2021-12-30

**Authors:** Arun Sharma, Rachel A. Clemens, Orquidea Garcia, D. Lansing Taylor, Nicole L. Wagner, Kelly A. Shepard, Anjali Gupta, Siobhan Malany, Alan J. Grodzinsky, Mary Kearns-Jonker, Devin B. Mair, Deok-Ho Kim, Michael S. Roberts, Jeanne F. Loring, Jianying Hu, Lara E. Warren, Sven Eenmaa, Joe Bozada, Eric Paljug, Mark Roth, Donald P. Taylor, Gary Rodrigue, Patrick Cantini, Amelia W. Smith, Marc A. Giulianotti, William R. Wagner

**Affiliations:** 1 Board of Governors Regenerative Medicine Institute, Cedars-Sinai Medical Center, Los Angeles, CA, USA; 2 Smidt Heart Institute, Cedars-Sinai Medical Center, Los Angeles, CA, USA; 3 Department of Biomedical Sciences, Cedars-Sinai Medical Center, Los Angeles, CA, USA; 4 Axiom Space, Inc., Houston, TX, USA; 5 Johnson & Johnson 3D Printing Innovation & Customer Solutions, Johnson & Johnson Services, Inc., Irvine, CA, USA; 6 University of Pittsburgh Drug Discovery Institute and Department of Computational and Systems Biology, University of Pittsburgh, Pittsburgh, PA, USA; 7 LambdaVision Inc., Farmington, CT, USA; 8 California Institute for Regenerative Medicine, Oakland, CA, USA; 9 Department of Pharmacodynamics, College of Pharmacy, University of Florida, Gainesville, FL, USA; 10 Departments of Biological Engineering, Mechanical Engineering and Electrical Engineering and Computer Science, Massachusetts Institute of Technology, Cambridge, MA, USA; 11 Department of Pathology and Human Anatomy, Loma Linda University School of Medicine, Loma Linda, CA, USA; 12 Department of Biomedical Engineering, Johns Hopkins University School of Medicine, Baltimore, MD, USA; 13 Department of Medicine, Johns Hopkins University School of Medicine, Baltimore, MD, USA; 14 Center for the Advancement of Science in Space, Inc, Melbourne, FL, USA; 15 Scripps Research Institute, San Diego, CA, USA; 16 Center for Computational Health IBM Research, Yorktown Heights, New York, NY, USA; 17 Joseph M. Katz Graduate School of Business, University of Pittsburgh, Pittsburgh, PA, USA; 18 Pittsburgh, PA, USA; 19 The Ohio State University, Columbus, OH, USA; 20 McGowan Institute for Regenerative Medicine, Pittsburgh, PA, USA; 21 Departments of Surgery, Bioengineering, Chemical Engineering, University of Pittsburgh, Pittsburgh, PA, USA

**Keywords:** microgravity, stem cells, microphysiological systems, organoids, biofabrication

## Abstract

Research in low Earth orbit (LEO) has become more accessible. The 2020 Biomanufacturing in Space Symposium reviewed space-based regenerative medicine research and discussed leveraging LEO to advance biomanufacturing for regenerative medicine applications. The symposium identified areas where financial investments could stimulate advancements overcoming technical barriers. Opportunities in disease modeling, stem-cell-derived products, and biofabrication were highlighted. The symposium will initiate a roadmap to a sustainable market for regenerative medicine biomanufacturing in space. This perspective summarizes the 2020 Biomanufacturing in Space Symposium, highlights key biomanufacturing opportunities in LEO, and lays the framework for a roadmap to regenerative medicine biomanufacturing in space.

## Teaser

Sharma and colleagues recap the 2020 Biomanufacturing in Space Symposium, which reviewed space-based regenerative medicine research and discussed opportunities to leverage low Earth orbit (LEO) to advance biomanufacturing for regenerative medicine. This perspective will highlight key biomanufacturing opportunities in LEO, note current technical gaps, and discuss next steps in developing a roadmap to biomanufacturing in space for regenerative medicine applications.

## Introduction

**Figure 1:**
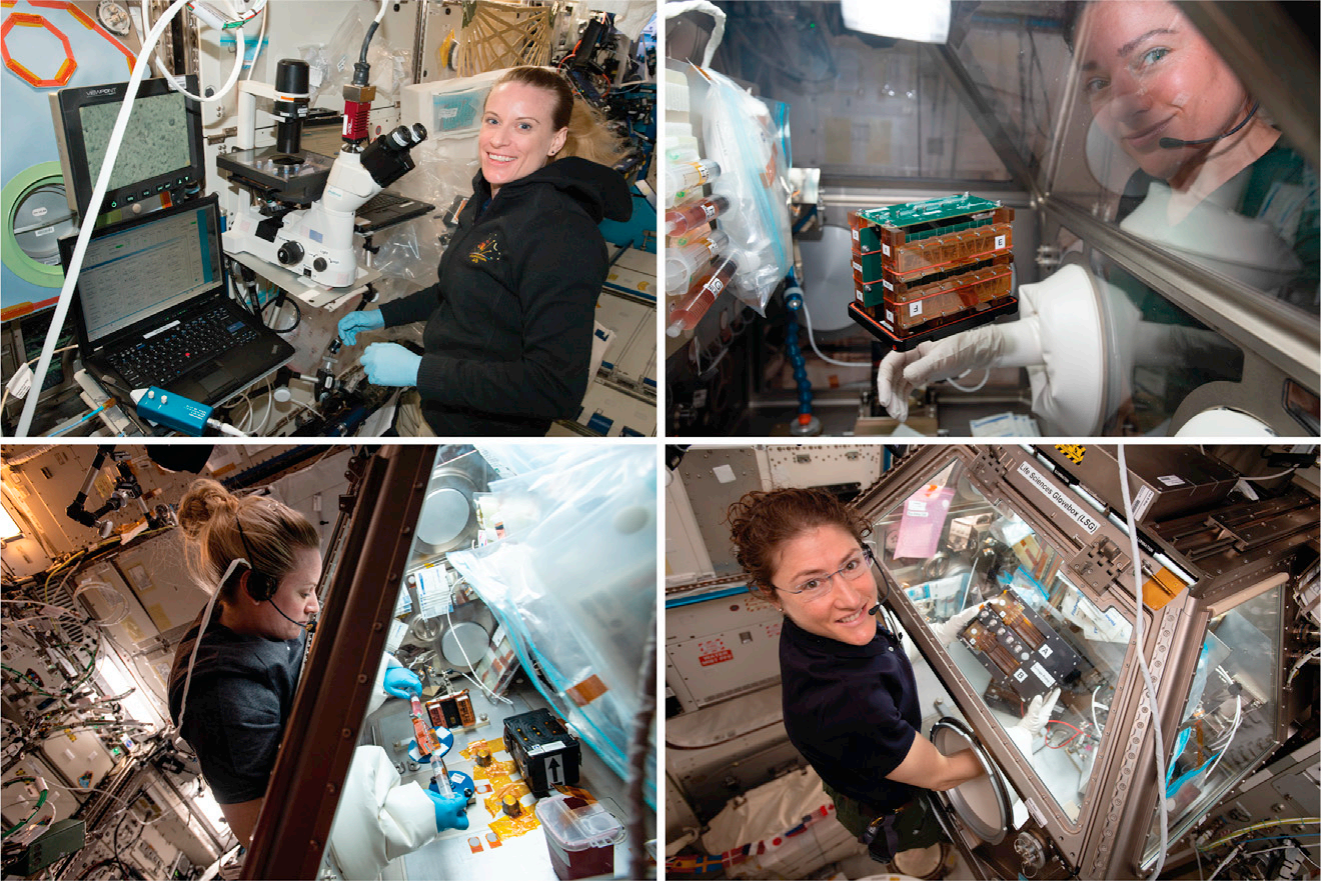
Examples of stem cell research aboard the ISS Top left: NASA astronaut Kate Rubins examines stem-cell-derived cardiomyocytes onboard the ISS. Top right: NASA astronaut Jessica Meir onboard the ISS working with engineered heart tissues. Bottom left: NASA astronaut Kate Rubins evaluates three-dimensional engineered heart tissue exposed to sustained microgravity conditions. Bottom Right: NASA astronaut Christina Koch examines a tissue chip system to study kidney function. Credit: NASA.

Over the last decade, the International Space Station National Laboratory (ISS National Lab) has supported space-based studies in the areas of tissue engineering and regenerative medicine (Figure 1). This initial research and development have provided important insights into how microgravity can be leveraged to advance biomanufacturing in space to benefit human life and commercial enterprise on Earth. Microgravity induces changes in bodily systems that result in effects including cardiovascular deconditioning, skeletal muscle atrophy, bone loss, and immune dysfunction, among others (Patel, 2020; Shelhamer et al., 2020; Smith, 2020; Vernikos and Schneider, 2010). These effects mimic the onset of health-related outcomes associated with aging and chronic human disease but at an accelerated rate. Such effects that could take years to manifest on Earth may develop in weeks in microgravity. While these changes are a concern for keeping astronauts safe on long-duration spaceflight, they also present an opportunity to study aging, disease progression, and test therapeutics on an accelerated timescale (Low and Giulianotti, 2019).

Utilizing microgravity has contributed to the collective fundamental knowledge of cellular behavior, cell-cell interactions, tissue development and regeneration, and aggregate interactions in the context of a whole organism (Bradbury et al., 2020; da Silveira et al., 2020; Garrett-Bakelman et al., 2019; Giulianotti and Low, 2019; Grimm et al., 2014; Herrmann et al., 2020). Pioneering bioengineering experiments on the ISS coupled with ground-based studies have demonstrated that microgravity enables the study of novel features not attainable under normal gravity conditions, including changes to stem cell proliferation rates and differentiation (Baio et al., 2018; Blaber et al., 2015; Imura et al., 2019; Jha et al., 2016; Yuge et al., 2006).

Additionally, bioprinting tissues in microgravity provides potential advantages for the use of lower viscosity biomaterials or bioinks and the ability to fabricate diaphanous biological structures. The processes involved in biofabrication are heavily reliant on biomechanical cues that are affected by gravity, and microgravity conditions should enable full control over these cues in ways not possible on Earth (Cubo-Mateo et al., 2020; Cubo-Mateo and Gelinsky, 2021; Moroni et al., 2021; Prasad et al., 2020; Swaminathan et al., 2021). Microgravity can also improve biofabrication processes that involve thin-layer deposition, through which thin film layers of biomaterial are deposited onto a substrate material with atomic-level precision. This could have significant value in the production of advanced medical devices.

### Current biomanufacturing research and development on the ISS

In recent years, the ISS has been increasingly more utilized by commercial, academic, and government users focused on leveraging microgravity for research and product development with Earth-based benefits (Giulianotti and Low, 2019; Parfenov et al., 2020). A number of government agencies have engaged in multiyear funding initiatives that utilize the ISS National Lab (Giulianotti and Low, 2019; Low and Giulianotti, 2019). For example, studies funded by the National Institutes of Health (NIH) and the National Science Foundation (NSF) have included space-based microphysiological systems (MPS) research to model kidney proximal and distal tubule physiology, cartilage-bone-synovium joint interactions, blood-brain barrier physiology, liver aging and immune response, and cardiac muscle tissue (Low and Giulianotti, 2019).

In addition, an increasing number of biotechnology companies are using the LEO environment to validate drug targets and test the efficacy of potential therapeutics on accelerated models of disease (Cadena et al., 2019; Giulianotti and Low, 2019). NASA and the ISS National Lab are working with a growing number of Commercial Service Providers, organizations that actively develop and operate ISS facilities and equipment that enables space-based biomedical research and development. In parallel, commercial companies are designing vehicles, satellites, and other platforms that will enable future opportunities in space-based biomanufacturing research and development. It is critical for stakeholders to work collaboratively to identify the best opportunities to utilize the ISS. Targeted research and development will enable a robust in-space regenerative medicine biomanufacturing market. Opportunities must be prioritized, such that the economic value of LEO-based research can be clearly demonstrated to then allow for further investment and growth based on this success.

## The Biomanufacturing in Space Symposium

CASIS and the McGowan Institute for Regenerative Medicine at the University of Pittsburgh hosted a Biomanufacturing in Space Symposium to serve as the first step in developing a roadmap to a sustainable market for biomanufacturing in space. The symposium included a series of virtual workshops, presentations, and interactive discussions with internationally recognized subject-matter experts in the areas of tissue engineering and regenerative medicine, as well as pioneers in space-based research and development. The goal was to identify the most promising opportunities to advance space-based biomanufacturing. Specifically, the opportunities identified needed to focus on the development and translation of commercially relevant biomolecules and biomaterials for use in pre-clinical, clinical, and therapeutic applications.

**Figure 2:**
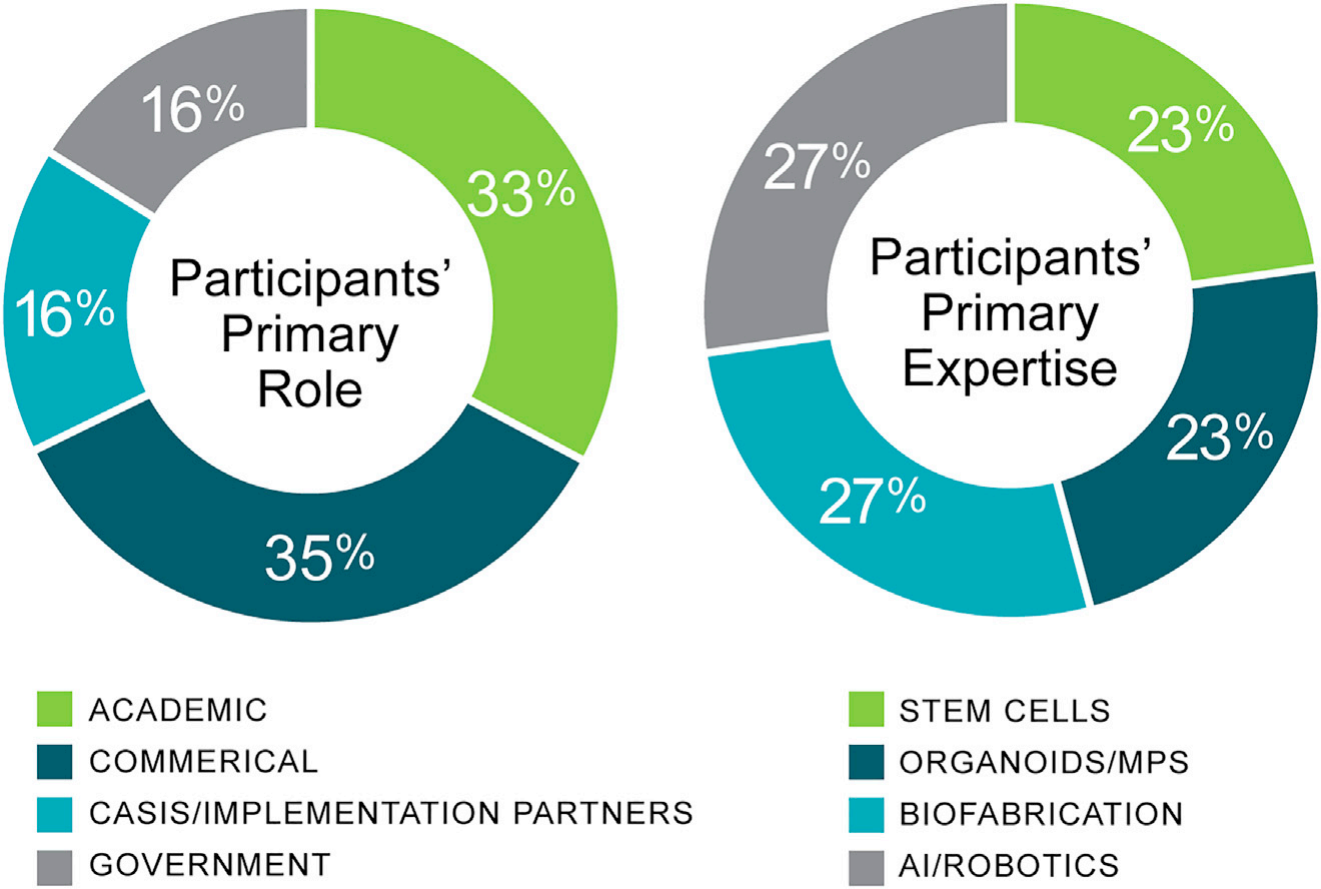
Breakdown of symposium participants’ expertise and primary role For those that identified stem cells as their primary expertise, 16 individuals’ primary role was academic (A), 5 were commercial (C), 5 were CASIS/Implementation Partners (CI), and 6 were government (G). Organoids/MPS had 12 A, 9 C, 5 CI, and 6 G. Biofabrication had 5 A, 17 C, 9 CI, and 6 G. AI/Robotics had 12 A, 17 C, 4 CI, and 4 G.

The Biomanufacturing in Space Symposium took place virtually in 2020 and 2021. The symposium’s 138 participants represented a diverse background of expertise, which significantly increased cross-education and learning (Figure 2). The symposium had three topic areas: stem cells, organoids and MPS, and biofabrication. Working sessions were divided into three phases: educate, generate, and prioritize.

The *educate phase* aimed to prepare symposium participants for interdisciplinary discussions via presentations by subject matter experts on the aforementioned topic areas and the stages to commercialization. Through a series of interactive sessions on each of the topic areas, the *generate phase* sought to identify the most promising opportunities to leverage the ISS for advancing space-based biomanufacturing and highlighted current knowledge gaps and commercial opportunities. In the *prioritize phase*, the key opportunities identified in each of the topic areas were refined and condensed, and the next steps in developing a roadmap for biomanufacturing in space were outlined. A separate symposium session explored methods for integrating automation, artificial intelligence (AI), and machine learning (ML) toward an agile iteration of scientific and research and development activities in LEO.

## Key opportunities identified

During the generate phase, more than 50 potential commercial opportunities were identified and prioritized based on the following criteria:

• Role of microgravity: The importance of microgravity in enabling the opportunity.
• Impact: The attractiveness of the opportunity for investment, including the magnitude of impact and concentration of investors.
• Risk: The risk associated with the opportunity, measured by the risk of failure, time to market, and risk-benefit tradeoffs.

The most promising opportunities identified through the symposium naturally codified into three areas: (1) disease modeling, (2) stem cells and stem-cell-derived products, and (3) biofabrication.

### Disease modeling

**Figure 3:**
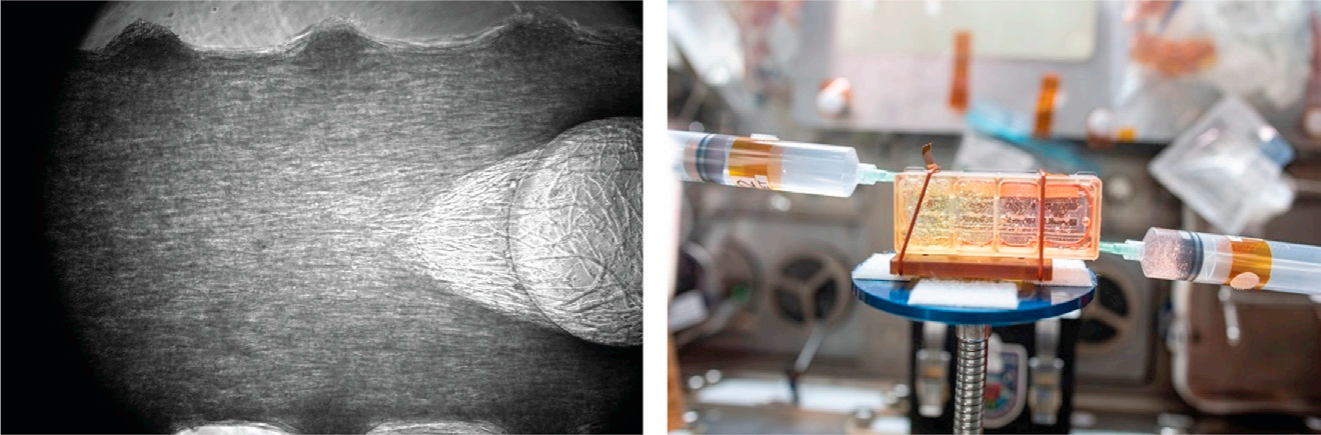
Examples of tissue engineering work aboard the ISS Left: engineered skeletal muscle tissue in a microfluidic chip in LEO, generated by Siobhan Malany Laboratory at the University of Florida in collaboration with Space Tango (credit: Siobhan Malany and Space Tango). Right: a NASA astronaut (out of frame) adds RNAlater reagent to a gas-permeable tissue chamber to preserve engineered heart tissue constructs for the Cardinal Heart investigation. Project led by Dr. Joseph Wu at Stanford University in collaboration with BioServe Space Technologies (credit: Joseph Wu and NASA).

Symposium participants extensively discussed the ability to utilize a sustained microgravity environment for disease modeling—whether through the use of stem cells, organoids, MPS platforms, or tissues either biofabricated in orbit or assembled terrestrially and brought to space (Figure 3). Participants highlighted the unmet need for novel approaches to model disease and aging (Low et al., 2021), and several researchers have utilized the ISS as a platform to develop disease models based on the physiological changes associated with spaceflight (Low and Giulianotti, 2019). Participants agreed that the opportunity to uniquely isolate the stresses induced by sustained microgravity could provide significant insights into the aging process and disease progression. Data from associated space-based studies indicate that humans experience significant physiological changes during adaptation to spaceflight and during readaptation upon return to Earth (Akima et al., 2000; Garrett-Bakelman et al., 2019; Luxton and Bailey, 2021; Meck et al., 2001).

#### Specific examples

##### Muscle wasting

Research has shown that during spaceflight, humans lose skeletal muscle at an accelerated rate, and countermeasures are required to dampen the accelerated loss (Vernikos and Schneider, 2010). Thus, microgravity-induced muscle loss provides an opportunity to study muscle-wasting progression on a faster timescale than is possible on Earth. Multiple studies have been conducted using rodents in microgravity as an accelerated disease model to elucidate mechanisms underlying muscle atrophy and to test new potential therapeutics (Chakraborty et al., 2020; da Silveira et al., 2020; Lawler et al., 2021; Semple et al., 2020; Smith et al., 2020). Moving beyond rodent models, microgravity provides a unique opportunity to study sarcopenia and disuse atrophy in human cellular models. Utilizing a LEO-based platform to study myocytes from different patient populations could enable the development of models for drug target identification and therapeutic evaluation. Such research could reveal druggable pathways that may not otherwise have been uncovered in terrestrial studies.

##### Changes in cardiac physiology

In microgravity conditions, humans experience acute changes in cardiac physiology, structure, and function. Long-term microgravity exposure leads to cardiac deconditioning. Due to this cardiac deconditioning, orthostatic intolerance is evident in astronauts upon returning to normal gravity (Lee et al., 2015). Arrhythmias have also occurred in astronauts during spaceflight, even in those with no prior history of arrhythmias (Delp et al., 2016). However, the acute cardiac changes associated with spaceflight largely return to baseline after return to normal gravity. Furthermore, studies indicate that astronauts on missions lasting 6 months to 1 year do not have an increased rate of developing cardiovascular disease after returning to Earth (Ade et al., 2017).

Thus, a LEO-based platform could enable the modeling of an acute, microgravity-induced cardiovascular phenotype and the reversal of this phenotype through the use of two-dimensional stem-cell-based models, three-dimensional tissue-engineered constructs, organoids, or MPS models. Preliminary work on the ISS (Figure 1) has demonstrated the successful culture and return of viable two-dimensional cardiomyocytes derived from induced pluripotent stem cells (iPSCs) and found that microgravity alters cardiac gene expression and function at the cellular level (Wnorowski et al., 2019). Additional studies are currently in progress utilizing multilineage, tissue-engineered cardiac constructs to study the impact of microgravity on cardiac physiology and function (Low and Giulianotti, 2019) (Figure 1). One of these studies uses an electroconductive decellularized extracellular matrix hydrogel that improves tissue maturation and function (Tsui et al., 2021).

##### Osteoarthritis

In microgravity conditions, humans experience accelerated bone loss, and extended spaceflight can alter bone integrity in a fashion roughly analogous to osteoporosis (Vico et al., 2000). In addition, the effects of altered loading of joint cartilage in space may affect the extent and rate of cartilage breakdown leading to osteoarthritis (Fitzgerald, 2017). The use of MPS or other tissue systems on a LEO-based platform could enable accelerated disease modeling, and such studies may provide unique insights into disease progression and uncover novel targets for therapeutic interventions to treat osteoarthritis. Furthermore, microgravity uniquely enables the study of disuse versus exercise in the management of osteoarthritis and post-traumatic osteoarthritis. Microgravity can enable studies using human cells and tissues to examine the effects of not only disuse but also applied mechanical force. Carefully controlled mechanical forces could be applied to tissues to mimic different types of exercise in conjunction with therapeutics. Studies are currently underway on the ISS utilizing a post-traumatic osteoarthritis MPS model to identify novel pathways to treat the condition and test therapeutic interventions (Low and Giulianotti, 2019).

##### Aging

Studies have shown that spaceflight induces several physiological changes in both astronauts and rodent models, including skeletal muscle atrophy, bone-density loss, immune dysfunction, cardiovascular deconditioning, and arterial stiffening, among others (Shen and Frishman, 2019). These changes, which resemble aging-related maladies on Earth, occur rapidly during spaceflight and are mostly reversible upon return to Earth. This provides a compelling case for leveraging microgravity conditions to improve the understanding of aging and related disease processes. MPS models, organoids, or biofabricated tissues exposed to microgravity and then returned to Earth could provide significant insight into novel biological targets associated with disease progression and regression as well as anti-aging.

Several space-based studies have been done on aging. One of the projects supported through the National Center for Advancing Translational Sciences (NCATS) Tissue Chips in Space initiative is leveraging an MPS model for immunological senescence in microgravity to investigate the relationship between immune aging and tissue healing as well as regenerative capacity (Low and Giulianotti, 2019). Additionally, NASA's One-Year Mission on the ISS with NASA astronaut Scott Kelly found that the average telomere length in Kelly's white blood cells increased during the mission and then returned to pre-flight levels after his return to Earth. However, in the months following his spaceflight mission, a greater number of his telomeres were lost or critically shortened (Garrett-Bakelman et al., 2019). These findings could present a therapeutic target that could be studied in the context of a LEO-based model, as telomere shortening and loss are associated with aging and susceptibility to age-related diseases, including cardiovascular issues and cancer.

##### Biofouling

Medical devices are prone to surface biofouling, which results from the nonspecific adhesion of proteins, cells, and microorganisms. These phenomena are associated with a substantial degree of morbidity and mortality across several types of implantable medical devices. Biofilms form on wetted surfaces during spaceflight (Zea et al., 2020), and on the ISS, the formation of microbial communities in the form of biofilms is often accelerated. A LEO-based platform could be used to accelerate the timeline for biofouling characterization of biomaterials and elucidate mechanisms that may be altered in microgravity in ways that increase or decrease virulence. In fact, studies have shown that during spaceflight, some bacterial strains appear to exhibit increased virulence (Simoes and Antunes, 2021).

#### Gaps

One of the primary current gaps noted during the symposium is the existing need for validation of space-based disease models for terrestrial applications. The question remains how clinically relevant the models are and how information obtained from the models may be utilized in therapeutic development (Low et al., 2021). These answers could be addressed in part by increased data and throughput, which were also noted as a current gap. The use of LEO for modeling terrestrial diseases is at a relatively nascent stage, and the ability to do large-scale experiments in the LEO environment is limited by the challenges of launching and conducting experiments in space. Symposium participants agreed that continued advancements in miniaturization, automation, the implementation of AI and machine learning (ML), and the standardization of equipment (hardware), biological materials, and protocols would increase the opportunity to generate meaningful data. Of note, these are all key areas where improvements in technologies for use in space will readily translate to and benefit terrestrial-based applications. Another key area for improvement that participants highlighted is the ability to rapidly iterate on experimental results, which is currently limited by the inherent logistical challenges of performing experiments in space. This gap could be addressed by having an inventory of in-orbit supplies such as banked cells and pre-seeded devices or organoids to provide the ability to manufacture models in space as needed. Additionally, increased throughput and data acquisition could be accelerated further as launch frequency continues to increase.

#### Commercial opportunities

One commercial opportunity identified during the symposium is in the data surrounding novel targets for therapeutic development. Participants discussed several ways in which this opportunity could be realized. The formation of a syndicate of pharmaceutical and biotechnology companies could serve to de-risk early stage common opportunities. This would lower the investment risk for any individual stakeholder and create the infrastructure for future single investments, with the tradeoff being the distributed control of any intellectual property developed through the syndicate. Another opportunity lies in collaborations with other government agencies and foundations that have interests in specific diseases that could be modeled in LEO. Participants also expressed enthusiasm about working with space agencies, commercial space companies, and other government agencies to find common areas that could inform both risk reduction in space exploration and advances in human health on Earth.

### More effective stem cells and stem-cell-derived products

Stem cells and stem-cell-derived products are promising as both research tools (Sharma et al., 2020) and therapeutic products (Ntege et al., 2020; Rodriguez-Fuentes et al., 2021; Sayed et al., 2016; Stern et al., 2018). Symposium participants discussed the potential of leveraging a LEO-based platform to gain insights into how to control and optimize stem cell pluripotency and multipotency, proliferation and expansion, genomic and epigenomic integrity, differentiation, and maturation. This opportunity area is supported by published work demonstrating that sustained microgravity influences the behaviors of stem cells and their derivatives.

#### Specific examples

##### Cells with increased potency and expansion capabilities

Stem cells are defined by their potency, or their ability to give rise to multiple derivative cell lineages. Pluripotent stem cells are able to transform into all cells of the body except for placental tissues. Multipotent stem cells can differentiate into cells of a specific lineage. However, a major challenge in the field is variability in stem cell potency from cell line to cell line, accompanied by an inability to maintain potency and genetic integrity as cells proliferate. Thus, it is critical to identify novel methodologies that will either maintain or enhance the potency, quality, and differentiation capacities of stem cell lines. Such improvements in cell characteristics would have an impact on the tissue engineering and regenerative medicine industries in both research and development and therapeutic applications. The symposium highlighted potential therapeutic applications that already have preliminary spaceflight data, including the following:

• **Creating cells and tissues for cardiac repair:** Following cardiac injury in the human heart, regeneration is limited (Bergmann et al., 2015). Methods to enable the proliferation of cardiomyocytes and subsequent cardiac regeneration are being actively investigated. Studies have found that cardiac progenitor cells cultured on the ISS exhibited increased proliferative and migratory potential due to changes in mechanotransduction pathways and, subsequently, cytoskeletal organization (Baio et al., 2018; Camberos et al., 2019). This is supported by studies using simulated microgravity on Earth (Jha et al., 2016). This initial work in sustained microgravity conditions indicates that such an environment can lead to the identification of novel targets for enhancing the therapeutic benefit of cardiovascular progenitor cells (Baio et al., 2018; Camberos et al., 2019, 2021).
• **Expansion of mesenchymal stem cells (MSCs) with improved clinical properties:** While MSCs hold potential for use as therapeutic agents, their safe and efficient expansion and appropriate characterization is still a major challenge in the field (Zhang et al., 2021). Recent spaceflight studies indicated that human MSCs can be grown safely on the ISS and that they have improved immunosuppressive capabilities compared with MSCs cultured on Earth (Huang et al., 2020). Additionally, MSCs cultured under simulated microgravity conditions showed increased therapeutic potential in a traumatic brain injury model (Otsuka et al., 2018).

##### Stem cell differentiation

Symposium participants also discussed the unique stimulus that a sustained microgravity environment can confer on stem cell differentiation, including the differentiation of iPSCs. The discussion ultimately centered on two primary themes: (1) what we could learn from an in-depth characterization of stem cell differentiation conducted in space, and (2) whether microgravity could allow for the generation of cell types not currently possible from terrestrially based *ex vivo* differentiation of stem cells, including the differentiation of iPSCs.

• **Characterizing stem cell differentiation in microgravity:** Terrestrial-based studies have demonstrated that differences in culturing conditions and cell source can have significant impacts on stem cell differentiation (Yim and Sheetz, 2012). While several studies have shown different effects to stem cells cultured in sustained microgravity, it is difficult to extrapolate results from these studies due to the incongruent nature of the conditions, equipment, and cell sources utilized. There is a need to fully characterize how microgravity as a variable influences stem cell differentiation into the three primary germ layers (mesoderm, ectoderm, and endoderm).

#### Gaps

During the symposium, the primary gaps identified for these opportunities centered around the current lack of data and standards. These are points in which much can be learned from terrestrial-based research efforts, where a lack of data and agreed-upon standards exist. To address these issues, the International Stem Cell Initiative (ISCI) has established standards for assessing the pluripotency of iPSCs (International Stem Cell, 2018), and the International Society for Stem Cell Research (ISSCR) has published guidelines to promote best practices (Daley et al., 2016). The community engaging in LEO-based stem cell research and development should align with current best practices in order to ensure space-based results are translatable across both spaceflight and terrestrial studies. Similarly, symposium participants discussed the importance of the origin and source of cells and the subsequent effects on results. Participants agreed that, in general, more than one donor source should be utilized, and the donor sources should be widely available (Mitchell et al., 2020). By adopting standards and applying high-throughput approaches, miniaturization, microfluidics, robotics, machine learning, and AI, the quality and quantity of data return could be dramatically amplified. Moreover, many of the technology advancements required to enable stem cell research on a LEO-based platform would readily translate to terrestrial applications, such as the development of compact, robust, high-throughput systems capable of working autonomously and remotely.

#### Commercial opportunities

Stem cells and stem-cell-derived products have two primary customer bases: (1) those who utilize stem cells as research tools, and (2) those who utilize stem cells in therapeutic applications. Symposium participants generally agreed that in the near term, the largest value return-on-investment will be derived from the data that can be translated to improve terrestrial processes, products, and tools. However, the participants felt that, ultimately, the large-scale production of certain types of stem cells and stem-cell-derived products could benefit from manufacturing in a LEO-based facility and that the LEO environment could confer certain advantages that may not be replicated in a terrestrial setting.

Symposium participants agreed that in the near term, this area of research would benefit from deliberate interactions with government agencies. The use of the unique LEO environment could provide key insights that may have substantial impacts to the larger research community and thus would be of great public interest. Publicly funded research and development is necessary to move beyond the current roadblocks and make commercial opportunities more attractive. As specific use cases emerge demonstrating that large-scale, in-space biomanufacturing provides the potential for a return on investment, increased commercial engagement will follow. To foster and prepare for these potential opportunities, discussions with cell-based therapeutic manufacturers, commercial space station developers, the research community, and the United States Food and Drug Administration (FDA) should begin now. These interactions should focus on the infrastructure needed to scale from LEO-based research facilities to LEO-based manufacturing facilities and on identifying the requirements needed to enable FDA approval.

### Biofabrication

During the symposium, biofabrication discussions covered a wide variety of opportunities, including fabricating tissues for disease modeling, testing and maturation of biofabricated materials, and improved fabrication processes for biomaterials and biofabricated constructs. With commercial companies having recently invested in technology development for terrestrial and in-space biofabrication, discussions at the symposium provided an overview of current efforts and a glimpse of future opportunities.

#### Specific examples

##### Thin-layer deposition

The process of assembling thin films through layer-by-layer deposition is of significant interest for applications such as optics, membranes, sensors, biomedicines, and several energy-related applications (Gentile et al., 2015; Richardson et al., 2015). In recent years, studies have focused on scaling up processes for thin-layer deposition to enable real-world applications (Richardson et al., 2015). Gravity-driven sedimentation of elements with differing densities can influence the speed at which new layers can be deposited as well as the ordering of molecular components into biologically functional assemblies. Sustained microgravity could be beneficial by potentially enhancing the production quality of products manufactured through thin-layer deposition. Reducing the influence of gravity-driven forces such as buoyancy and sedimentation on the surface tension and homogeneity of the solutions or materials used for deposition could allow for more uniform layering and a higher-quality multilayer (Dag et al., 1997). In medical applications, these benefits could provide improved ductility for alloys in devices such as stents.

##### Tools for biofabrication

There is an urgent need for tissues, organs, and other biomaterials for use in transplantation and regenerative medicine applications (Hunsberger et al., 2020; Ntege et al., 2020). In the past decades, several novel biofabrication tools have emerged to enable the assembly of complex structures (Pedde et al., 2017). All of these tools either integrate or attempt to circumvent the effects of gravity in their biofabrication processes. Given the broad demand for additional biofabrication tools and techniques, discussions during the symposium centered on potential advantages that biofabrication in space might confer (Moroni et al., 2021). For example, sustained microgravity could enable the use of less viscous bioinks and reduce the reliance on chemical and physical cross-linking strategies for rapid structural stabilization that must be applied in concert with printing. Microgravity could also provide the ability to enhance cell-cell interactions for organoid production, the ability to control mechanotransduction effects due to gravity during the maturation/curing process, and the ability to print simultaneously from any spatial orientation.

#### Gaps

Biofabrication is a relatively recent addition to the ISS research portfolio. Early identification of gaps will support effective guidance toward a more efficient development of the field. However, the challenge is to do so in a way that also leaves room for innovation and discovery. Initial discussions regarding gaps centered on the need to gain additional insights on fundamental behaviors of materials (i.e., cells, liquids, and proteins) in a sustained microgravity environment and to determine how such information could influence the design and utilization of biofabrication approaches (Ahari et al., 1997; Dag et al., 1997; McPherson and DeLucas, 2015). Additionally, given that biofabrication processes would occur remotely (on unmanned platforms in LEO) with communication delays of many seconds, the need for robust automation linked to machine learning was also a focal point of discussion.

#### Commercial opportunities

Symposium participants identified two primary opportunities to enable the further development of space-based biofabrication applications: public funding and private investment. The collective need for novel approaches to produce implants, tissues, and organs is a public concern, and there are opportunities for government agencies to put resources into utilizing a unique environment such as a LEO-based platform to advance the field. For applications where commercial opportunities are identified, symposium discussions centered on private investments and the challenges around investors’ desire for short timelines and a multiplier for a return on investment. Participants agreed that private investments in specific applications had potential if the risk-versus-return valuations could satisfy investors. As more commercial companies become involved in developing spacecraft and in-orbit platforms, there will likely be a continued reduction in the costs and time associated with LEO-based manufacturing.

## Automation, artificial intelligence, and machine learning

**Figure 4:**
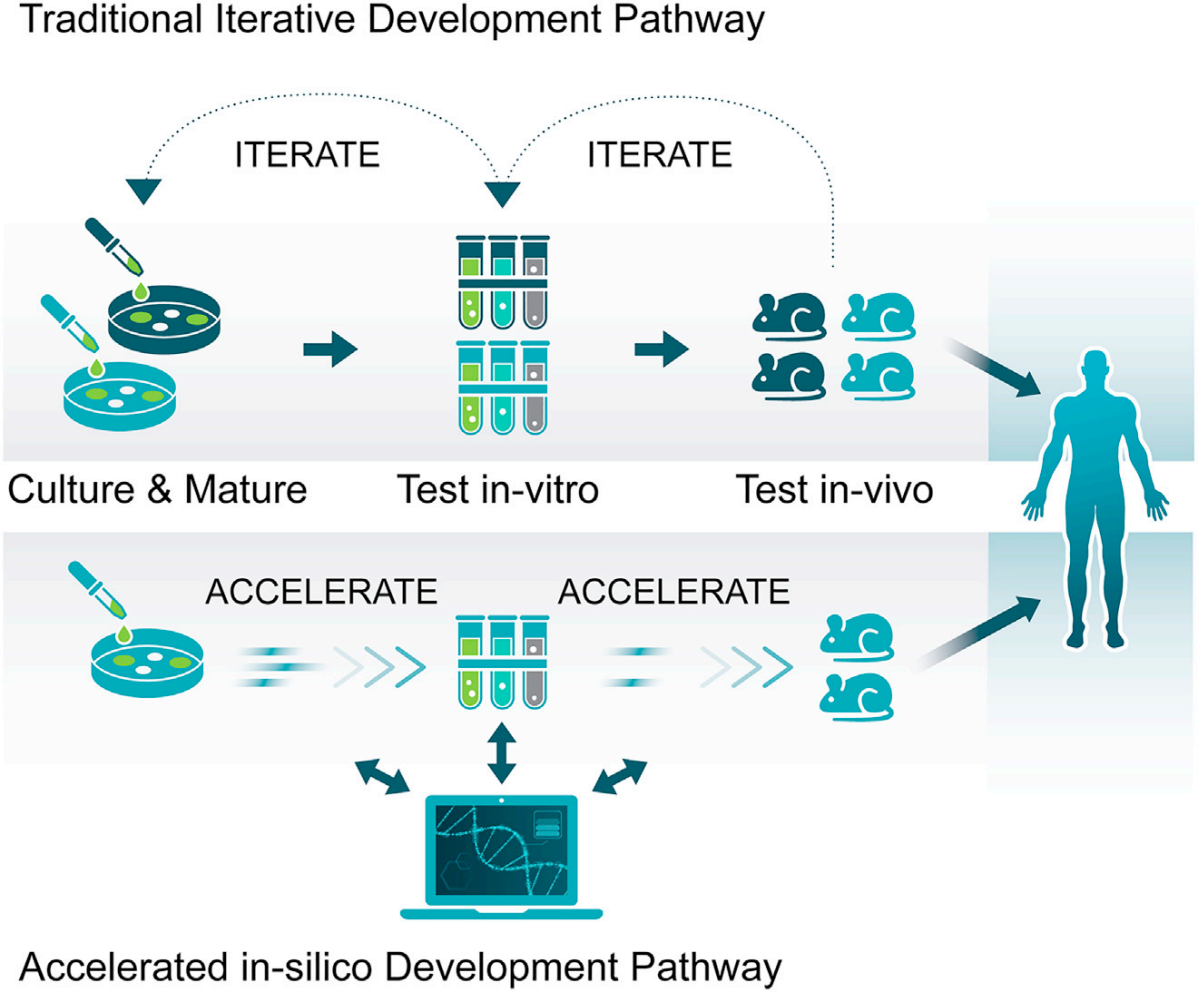
Evolution of therapeutic discovery, testing, and translation pathways Development pathways integrated with automation, machine learning, and artificial intelligence can accelerate the process and utilize fewer resources

The need for more data points to substantiate the identity and validity of the most valuable scientific and commercial opportunities that the LEO environment confers was a common theme that emerged within working groups throughout the symposium. The current lack of data is partly due to the challenging nature of accessing LEO and conducting research in a space-based environment. Symposium participants consistently noted that utilizing advances in automation, AI, and ML could enable an exponential production of the data needed to make informed decisions downstream (Figure 4).

Currently, tools are being developed to automate terrestrial cell culture (Cohen-Karlik et al., 2021) and biofabrication methods (De Pieri et al., 2021). These technologies could be applied to research in LEO, allowing experiments to run autonomously and potentially scale in progression from research to clinical applications (Vieira et al., 2021). This approach would enable researchers to continuously generate data and test conditions in a manner that reduces the need for astronaut intervention and associated astronaut training. Automation would also facilitate intellectual property protection, as closed systems could be devised to protect confidential or proprietary work steps.

Additionally, research groups are applying ML and AI to improve cellular products (Cohen-Karlik et al., 2021; Mota et al., 2021), biomaterial manufacturing (An et al., 2021; Lee et al., 2020), and disease modeling (Severson et al., 2021). Utilizing existing datasets, both from terrestrial experiments as well as LEO-based experiments (da Silveira et al., 2020), ML approaches could be built into the automated LEO platforms. As new data are generated, more advanced AI approaches could be utilized to focus on the specific applications where LEO confers advantages over terrestrial-based facilities.

## Market analysis

Rapid advancements are being made in space infrastructure technologies that provide increased frequency and low costs for accessing space. Thus, there is an increase in business plans from companies aiming to build free fliers and modular commercial space stations available to a diverse set of users. Development of such LEO-based platforms will build on lessons learned from the ISS and will enable space-based research and development and manufacturing capabilities that will provide an infrastructure for the further maturation of future business cases and models. To assess the financial impact that space-based biomanufacturing could have on the utilization of such infrastructure and the evolution of a commercial LEO economy, we performed a preliminary market size analysis.

**Figure 5:**
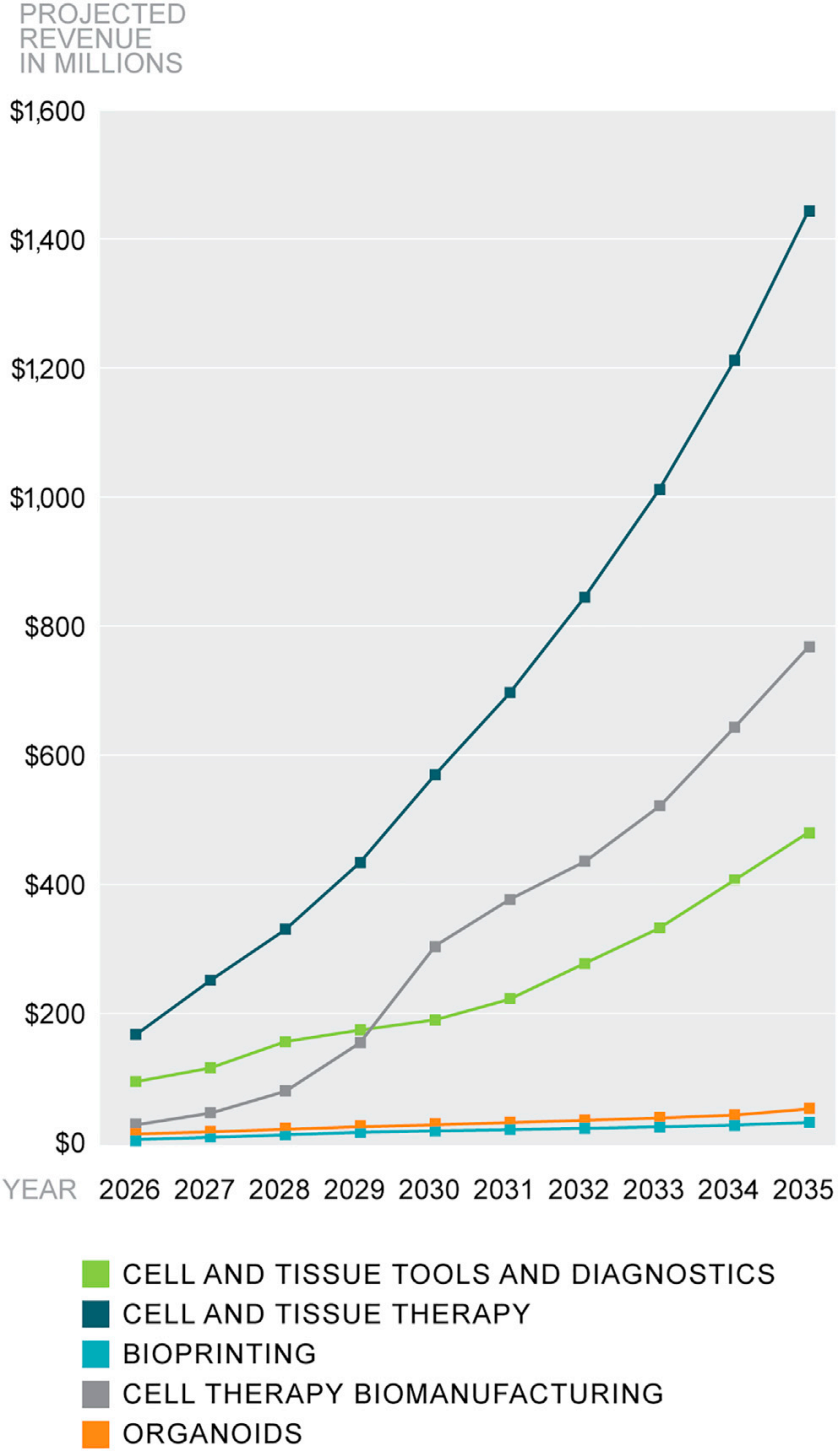
Biomanufacturing in low Earth orbit market subsegmentation revenue projection The LEO biomanufacturing market is broken into five primary subsegments: (1) cell and tissue tools and diagnostics, (2) cell and tissue therapy, (3) bioprinting, (4) cell therapy biomanufacturing, and (5) organoids. Projections are for the next 15 years.

It is important to note that most biomanufacturing technologies discussed here, while potentially addressing disruptive opportunities, have very significant early stage innovation and development risks. Thus, we have chosen to present our estimates as a relatively conservative scenario (Figure 5). If a disruptive technology with positive economic potential is successfully developed, the product-specific growth rates that could be achieved could far exceed the compound annual growth rate metrics discussed here. We segmented the LEO biomanufacturing market into five primary subsegments: (1) cell and tissue tools and diagnostics, (2) cell and tissue therapy, (3) bioprinting, (4) cell therapy biomanufacturing, and (5) organoids.

For the preliminary market sizing analysis, market research reports were queried using the online University of Pittsburgh Library System and a general internet search. Market research databases included BCC Research, IBISWorld, and Transparency Market Research. Market data from these research reports were grouped by the five primary market subsegments. The cell and tissue tools and diagnostics market were further subsegmented into seven markets: (1) cell line, (2) cell harvesting, (3) cell expansion, (4) cell and gene therapy tools and reagents, (5) cancer cell analysis, (6) cell viability assays, and (7) cell-based assays. The cell and tissue therapy market was subsegmented into four markets: (1) tissue engineering and regeneration, (2) stem cell and regenerative therapy, (3) iPSCs, and (4) cell therapy processing.

## Developing a roadmap to biomanufacturing in space for regenerative medicine

Biomanufacturing in space has potential to enable scientific and technological advancements not achievable on Earth, leading to products providing both economic value and Earth-side benefits. However, to develop a sustainable market in LEO, additional targeted research and development is required to demonstrate the viability and economic value of space-based biomanufacturing. The Biomanufacturing in Space Symposium was the first step in identifying and prioritizing the key opportunities to pursue. Symposium participants concluded that establishing a public-private consortium was the best way to advance these opportunities toward the development of a biomanufacturing marketplace in LEO. Continued public-sector funding is crucial to further de-risk space-based research and development and facilitate investment and market growth. Private-sector involvement is also essential to guide the research and development to ensure it is focused on key marketplace needs. A public-private consortium would serve to further prioritize and de-risk space-based biomanufacturing research and development and translate results into commercial products for use in pre-clinical, clinical, and therapeutic regenerative medicine applications on Earth.

The next steps of a public-private consortium are: (1) develop a structure and governance model to expedite the development and translation of biomanufacturing in LEO, (2) establish an integrated process outlining the role of the consortium from discovery to commercialization of a LEO-based product, and (3) recruit members for the consortium. The structure for the consortium should include an oversight board that sets priorities, provides resources, manages knowledge capture, and serves as a single point of contact for membership and external stakeholders. The board should include advisory committees of experts in three areas: industry/commercial, scientific/clinical, and LEO-based operations.

The Consortium should follow a staged commercialization process that begins with a discovery/concept stage and culminates in a product to market. The project/product must achieve specific activities and critical milestones as pre-defined for each stage before advancing to the next stage. The oversight board and advisory committees will be comprised of representatives from five key groups: (1) commercial implementers (from pharmaceutical companies, contract development and manufacturing organizations, etc.), (2) technology developers from universities, institutes, and research and development organizations, (3) technology enablers with a focus on AI, robotics, and automation, (4) launch and payload operations experts, and (5) public agencies (e.g., science, space, defense, and regulatory agencies).

Commercialization for any new biomedical platform will take time, and the Consortium could be defined by four phases that, taken together, span 10 years: (1) business foundation development, (2) research identification, prioritization, and development, (3) technology translation, approval, and application, and (4) technology manufacturing and commercialization. Based on discussions at the symposium, many key opportunities for biomanufacturing in space are in the second phase.

## Conclusions

The last two decades have seen remarkable advances in regenerative medicine and exponential advancement in space technologies, enabling new opportunities to access and commercialize space. The Biomanufacturing in Space Symposium assembled thought leaders and experts to identify promising opportunities, current gaps, and pathways to realizing the full potential of LEO for biomanufacturing. It is time to leverage LEO to conduct research and development that demonstrate the value of space-based biomanufacturing and its benefits to humankind. This will enable the investments required for a robust biomanufacturing market in space, and this symposium was a first step towards developing this future.

## Author contributions

Conceptualization, M.A.G., G.R., P.C., W.R.W., and A.S.; methodology, M.A.G., G.R., P.C., W.R.W., A.S., J.B., and E.P.; validation, M.A.G., G.R., P.C., W.R.W., J.B., E.P., and A.S.; formal analysis, M.A.G., G.R., P.C., W.R.W., J.B., E.P., D.P.T., and A.S.; resources, M.A.G., R.A.C., O.G., D.L.T., N.L.W., K.A.S., A.J.G., S.M., A.G., M.K.-J., D.B.M., D.-H.K., J.F.L., M.S.R., J.H., L.E.W., J.B., E.P., M.R., D.P.T., G.R., P.C., A.W.S., W.R.W., and A.S.; writing, M.A.G., R.A.C., O.G., D.L.T., N.L.W., K.A.S., A.J.G., S.M., A.G., M.K.-J., D.B.M., D.-H.K., J.F.L., M.S.R., J.H., L.E.W., S.E., J.B., E.P., M.R., D.P.T., G.R., P.C., A.W.S., W.R.W., and A.S.; visualization, M.A.G., E.P., D.P.T., G.R., and A.W.S.; supervision, M.A.G., W.R.W., and A.S.; project administration, M.A.G., G.R., P.C., W.R.W., and A.S.; funding acquisition, M.A.G.

## Conflict of interests

R.A.C. and A.G. are employees of Axiom Space, Inc. N.L.W. is an owner and employee of LambdaVision, Inc.
